# Navigating end-of-life decision-making in nursing: a systematic review of ethical challenges and palliative care practices

**DOI:** 10.1186/s12912-024-02087-5

**Published:** 2024-07-09

**Authors:** Majed Awad Alanazi, Marwa Mamdouh Shaban, Osama Mohamed Elsayed Ramadan, Mohammed Elsayed Zaky, Huda Hamdy Mohammed, Fatma Gomaa Mohamed Amer, Mostafa Shaban

**Affiliations:** 1 College of Nursing, Jouf University, Sakaka, Al Jouf, 72388 Saudi Arabia; 2 Community Health Nursing- Faculty of Nursing- Cairo University, Cairo, Egypt; 3 Faculty of Nursing- Cairo University, Cairo, Egypt

**Keywords:** Nursing ethics, End-of-life decision-making, Palliative care, Communication, Education

## Abstract

**Objectives:**

This systematic review aimed to synthesize evidence on the ethical dilemma’s nurses encounter in end-of-life care and effective palliative care practices. The objectives were to understand key ethical issues, evaluate communication and decision-making strategies, and identify approaches to support nurses and patients.

**Methods:**

A comprehensive search of major databases was conducted according to the PRISMA guidelines. Studies directly relating to nursing ethics, challenges in end-of-life decision-making, and palliative care practices were included. The risk of bias was assessed using ROBVIS-II. Data on ethical issues, palliative interventions, and outcomes was extracted and analyzed thematically.

**Results:**

22 studies met the inclusion criteria. Key themes that emerged were: (1) Effective communication and involving patients in decision-making are essential but complex. (2) Nurses face dilemmas around balancing autonomy, beneficence and relational issues. (3) Integrating palliative care principles enhances symptom management and aligns care with patient values. (4) Education and organizational support are needed to equip nurses with skills and coping strategies.

**Conclusion:**

Navigating end-of-life care requires addressing interconnected ethical, communication and support needs. While studies provided insights, further research is required on cultural competence training, standardized education programs and longitudinal evaluations.

**Supplementary Information:**

The online version contains supplementary material available at 10.1186/s12912-024-02087-5.

## Introduction

End-of-life decision-making presents complex ethical challenges for patients, families, and healthcare providers. As medical technologies advance, patients are living longer with serious illness, and end-of-life care planning is becoming increasingly important [1]. Palliative care practices aim to improve quality of life for patients with serious illness through pain and symptom management, while also providing support for difficult medical decisions near the end of life [2]. This review aimed to provide background on key ethical issues in end-of-life decision-making and summarize evidence on communication strategies, advance care planning interventions, and palliative care models to support patients and families navigating this difficult process.

A fundamental ethical principle in end-of-life care is respect for patient autonomy – the idea that patients have the right to make their own healthcare decisions consistent with their values and priorities [3]. However, many factors can impede patient autonomy. These include acute changes in decision-making capacity from illness progression, family/provider conflicts about appropriate goals of care, and racial/ethnic/cultural differences in end-of-life decision-making norms [4]. Surrogate decision makers for incapacitated patients may struggle to balance substituted judgment (making decisions aligned with the patient’s known preferences) and best interest standards (choosing options that seem to maximize benefit and minimize harm based on available evidence) [5, 6]. Palliative sedation is an ethically accepted practice aimed at relieving intractable suffering in end-of-life care without hastening death. With competent practices, the risk of death due to palliative sedation is highly unlikely, aligning with the ethical principle of non-maleficence [7]. The goal is always to relieve suffering while maintaining patient dignity [8].

Clear communication and conflict resolution strategies are needed to address these ethical complexities [9]. Various communication models aim to support truly shared decision-making near the end of life [10]. Principle-based approaches emphasize upholding patient autonomy through informed consent. Welfare-based models focus more on determining objective best interests by balancing benefits and burdens of treatment options [11]. Relationship-based perspectives highlight the importance of shared deliberation, surfacing unspoken needs and sustaining family relationships [12]. Evidence suggests relationship-based approaches may have advantages in end-of-life communication, as they allow space for emotional catharsis and subtle negotiation of values while still respecting patient priorities [13].

Numerous structured communication interventions also show promise for improving the quality of end-of-life conversations and reducing conflict in diverse care settings ranging from ICUs to nursing homes [14]. For example, the VitalTalk model uses role-play and feedback to train clinicians in core communication skills to promote goal-concordant care for seriously ill patients [15]. However, major communication challenges often persist near the end of life, especially regarding conveying prognosis and explaining care limitations for patients with severely compromised health [16]. Cultural factors further complicate communication norms, as some patients and families from non-Western backgrounds may believe discussing death directly can be harmful or show disrespect. More research is needed to adapt end-of-life communication training approaches to cultural contexts [17].

Advance care planning represents another vital component of ethical end-of-life care, providing a process for patients to document preferences to guide treatment decisions in the event of future incapacity [18]. However, studies suggest fewer than one third of American adults have completed any kind of advance directive. Key barriers include difficulty contemplating one’s own mortality, lack of public awareness, time constraints with healthcare providers, and concerns that advance directives may undermine hope or be misinterpreted later [19]. A range of advance care planning interventions have shown promise in increasing documentation of patient preferences and care aligned with patient values and goals [20]. Notable examples include video-based decision aids to assist completion of living wills and Portable Medical Orders (POLST) forms, which provide specific medical orders for compromised patients [21].

Research finds POLST forms are substantially more effective than traditional, more vague living wills on their own in ensuring patient preferences such as DNR orders are actually honored [22]. However, additional research is needed on cultural differences in values and preferences around advance care planning, as well as dissemination strategies to increase access to POLST paradigm programs [23]. Further study should also explore potential ethical downsides of POLST forms, including concerns regarding self-determination if patients feel pressured into unwanted orders, or possible over-emphasis on technical treatment options rather than eliciting patient values and priorities [24].

In addition to advance care planning, palliative care models aim to improve quality of life and provide multifaceted support for patients and families facing serious illness through comprehensive symptom management, psychological and spiritual care, and coordination of goals and values with treatment options across care settings [25]. The End of Life Nursing Education Consortium (ELNEC) provides comprehensive training and resources for nurses to deliver quality end-of-life care. ELNEC’s curriculum includes critical aspects such as pain management, ethical issues, cultural competence, and communication strategies, which are essential for enhancing nurses’ competencies in palliative care [26]. Numerous studies demonstrate benefits of early, integrated palliative care provided alongside disease treatment for conditions like advanced cancer. These benefits include improvements in quality of life, reduced rates of depression, more appropriate referrals to hospice, and even survival gains for certain cancer patients [27, 28]. However, major access barriers persist. Significant racial, ethnic, and rural disparities remain in utilization of specialist palliative care and hospice services [29].

Novel models like home-based palliative care programs may help increase access for underserved populations, while telehealth options can also extend services to remote areas [30]. However, more research is needed on potential technological barriers and privacy risks of virtual palliative care. Additionally, palliative care historically has focused predominantly on cancer populations, while evidence on optimal models tailored to other terminal illnesses like end-stage organ failure and neurodegenerative conditions remains limited [31]. For example, nursing homes have some of the highest death rates yet variable integration of palliative care principles. Quality of end-of-life care in nursing homes depends heavily on staff education in symptom management, nursing home resources, and state regulatory policies on palliative approaches [32]. As nursing homes disproportionately serve minorities already at high risk for undertreatment of pain and other symptoms, enhancing palliative care in this setting could help address care disparities [33]. More implementation research should investigate multifaceted strategies to integrate palliative care principles into nursing homes, such as staff skills training, facility culture changes, and policy initiatives [34].

Looking beyond current practices, recent studies have addressed many of the complexities surrounding end-of-life care, including ethical decision-making, communication strategies, and cultural competence. These advancements highlight the need for continuous updates and adaptations in palliative care practices [35]. . Although early outpatient specialty palliative care reduces ICU admissions at the end of life, further study is needed to clarify net impacts on healthcare spending across multiple settings, which could inform policies on coverage and payment incentives for palliative care [36]. Many nurses also report feeling unprepared and overwhelmed when discussing end-of-life preferences and providing care to seriously ill patients [37]. Investigating associations between palliative care integration and clinician stress, career satisfaction, and retention may highlight benefits to healthcare staff as well as patients [38].

Finally, robust evidence on cultural competence interventions is needed to address significant disparities in access to and experiences with end-of-life care among minority groups [39]. Racial and ethnic minorities are less likely to complete advance directives, more likely to prefer life-prolonging measures at end of life, and report lower quality end-of-life care compared to white Americans [40]. Culturally tailored advance care planning and communication approaches could help reduce these disparities. Diversifying the palliative care and hospice workforce is also critical to bringing cultural humility to end-of-life services [41].

Delivering ethical, patient-centered end-of-life care requires aligning treatments with patient values and goals through skillful communication and informed advance care planning, which must account for cultural factors [42]. Evidence-based palliative care models integrate physical comfort and holistic support with shared decision-making grounded in patient priorities. However, major gaps remain in access to quality end-of-life care [43]. Further research on communication interventions, advance directive dissemination, palliative care integration across diverse settings and populations, impacts on costs and clinicians, and reducing disparities can help support patients and families navigating high-quality, ethical care through this challenging yet inevitable phase of the healthcare journey [44].

Despite extensive research on ethical decision-making and palliative care practices, significant gaps remain. Firstly, there is a lack of comprehensive understanding of how cultural differences impact end-of-life decision-making, particularly in non-Western contexts. Secondly, the integration of palliative care principles into various healthcare settings, especially in regions with limited access to specialized palliative care services, requires further exploration. Thirdly, there is a need for more targeted education and training programs to equip nurses with the skills necessary to navigate complex ethical dilemmas and provide culturally competent care. This systematic review aims to address these knowledge gaps by synthesizing current evidence on ethical challenges and palliative care practices, identifying effective communication and decision-making strategies, and proposing areas for future research to enhance end-of-life care for diverse populations.

## Method

### Search strategy and selection criteria

This systematic review strictly adheres to the Preferred Reporting Items for Systematic Reviews and Meta-Analyses (PRISMA) guidelines, ensuring adherence to the highest standards of rigor and transparency. Following the PRISMA Protocols (PRISMA-P) statement, we developed a detailed research protocol, which was registered with PROSPERO (CRD42024513767), demonstrating our commitment to methodological precision and integrity.

Our literature search strategy was designed to be comprehensive, spanning multiple prestigious databases: Embase.com (Scopus), Medline ALL (Ovid), CINHAL, Web of Science Core Collection, Cochrane Central Register of Controlled Trials (Wiley), and Google Scholar. This approach aims to encompass the breadth and depth of existing research on end-of-life decision-making and palliative care practices within nursing. The last search, conducted on 17-1-2024 (which started from 2000 to 2024), We have revised our search terms to include the correct MeSH terms such as ‘Terminal Care,’ ‘Palliative Medicine,’ ‘Hospice and Palliative Care Nursing,’ ‘Ethics,’ and ‘Hospice Care.’ This ensures a more robust methodological approach.

Keywords and search terms were chosen to capture the complexities of ethical dilemmas nurses face in end-of-life care, strategies for addressing these challenges, and the implementation of palliative care practices. This targeted search strategy aimed to comprehensively review literature on the ethical dimensions of end-of-life care decision-making in nursing, including the perspectives, experiences, and practices of nursing professionals in various healthcare settings (Table 1).

The selection criteria for studies included in this review will focus on qualitative, quantitative, and mixed-methods research that directly addresses ethical challenges and palliative care practices in nursing, with particular emphasis on studies that offer insights into effective communication, decision-making processes, and the application of ethical principles in end-of-life care. Priority will be given to peer-reviewed articles, guidelines, and consensus statements that contribute significantly to understanding and improving ethical decision-making and palliative care practices in nursing.

**Table 1:** Search strategy

Database	Search Terms
**PubMed**	‘Terminal Care OR Palliative Care OR ‘Hospice and Palliative Care Nursing AND Ethics Nursing OR Ethical Dilemmas OR Decision Making AND Nursing OR Nurses OR Nursing Care
**MEDLINE**	Same as PubMed
**Embase**	(‘Terminal Care /exp OR ‘palliative care’ OR ‘terminal care’) AND (‘Hospice and Palliative Care Nursing ‘/exp OR ‘ethical dilemmas’ OR ‘decision making’) AND (‘nursing’/exp OR ‘nurses’ OR ‘nursing care’)
**Web of Science**	TS = (‘Terminal Care OR palliative care OR terminal care) AND TS = (ethics, nursing OR ethical dilemmas OR decision making) AND TS = (nursing OR nurses OR nursing care)
**Cochrane Library**	“’Terminal Care " OR “Palliative Care” OR “Terminal Care” AND “’Hospice and Palliative Care Nursing " OR “Ethical Dilemmas” OR “Decision Making” AND “Nursing” OR “Nurses”
**Google Scholar**	(“’Terminal Care Care” OR “Palliative Care” OR “Terminal Care”) AND (“Ethics, Nursing” OR “Ethical Dilemmas” OR “Decision Making”) AND (“Nursing” OR “Nurses”)

### Eligibility criteria for screening

Following the removal of duplicates from our search output, we conducted a preliminary screening of titles and abstracts, leading to a thorough evaluation of full-text articles. Our inclusion criteria were designed to identify publications such as original research articles, and clinical trials involving human subjects. The primary focus was on studies that addressed ethical challenges and palliative care practices in the nursing domain, specifically within the context of end-of-life decision-making. We sought research that provided insights into ethical dilemmas, strategies for navigating these challenges, approaches to palliative care, and the role of nurses in managing end-of-life care. This included studies that examined the effectiveness of communication strategies, decision-making processes, advance care planning, and the impact of ethical training on nursing practice. We prioritized studies that offered comparative analyses of different ethical approaches, palliative care models, and interventions aimed at supporting nurses and patients through the end-of-life care journey. The key outcomes of interest were improvements in ethical decision-making, patient-nurse relationships, quality of end-of-life care, and nurse well-being.

Exclusion criteria were applied to case reports, case series, publications only available as abstracts, letters, editorials, and conference materials, as well as studies conducted on animals or in vitro. We also excluded research not explicitly focusing on nursing practices related to end-of-life care or ethical challenges, studies that did not implement specific interventions aimed at improving palliative care or ethical decision-making in nursing, and those lacking in-depth methodological detail. Research that adhered strictly to existing ethical or palliative care guidelines without introducing novel approaches, as well as studies without control groups or insufficient data for meaningful analysis, were omitted. Additionally, studies failing to offer new insights into ethical or palliative care practices in nursing or published in languages other than English without accessible translations were disregarded. These exclusion criteria ensured our systematic review concentrated on pertinent, high-caliber research that significantly advances understanding of ethical and palliative care practices in nursing, particularly at the end of life, thereby aligning our investigation with the broader research objectives and contributing to the existing literature on this critical topic.

**Fig. 1:**
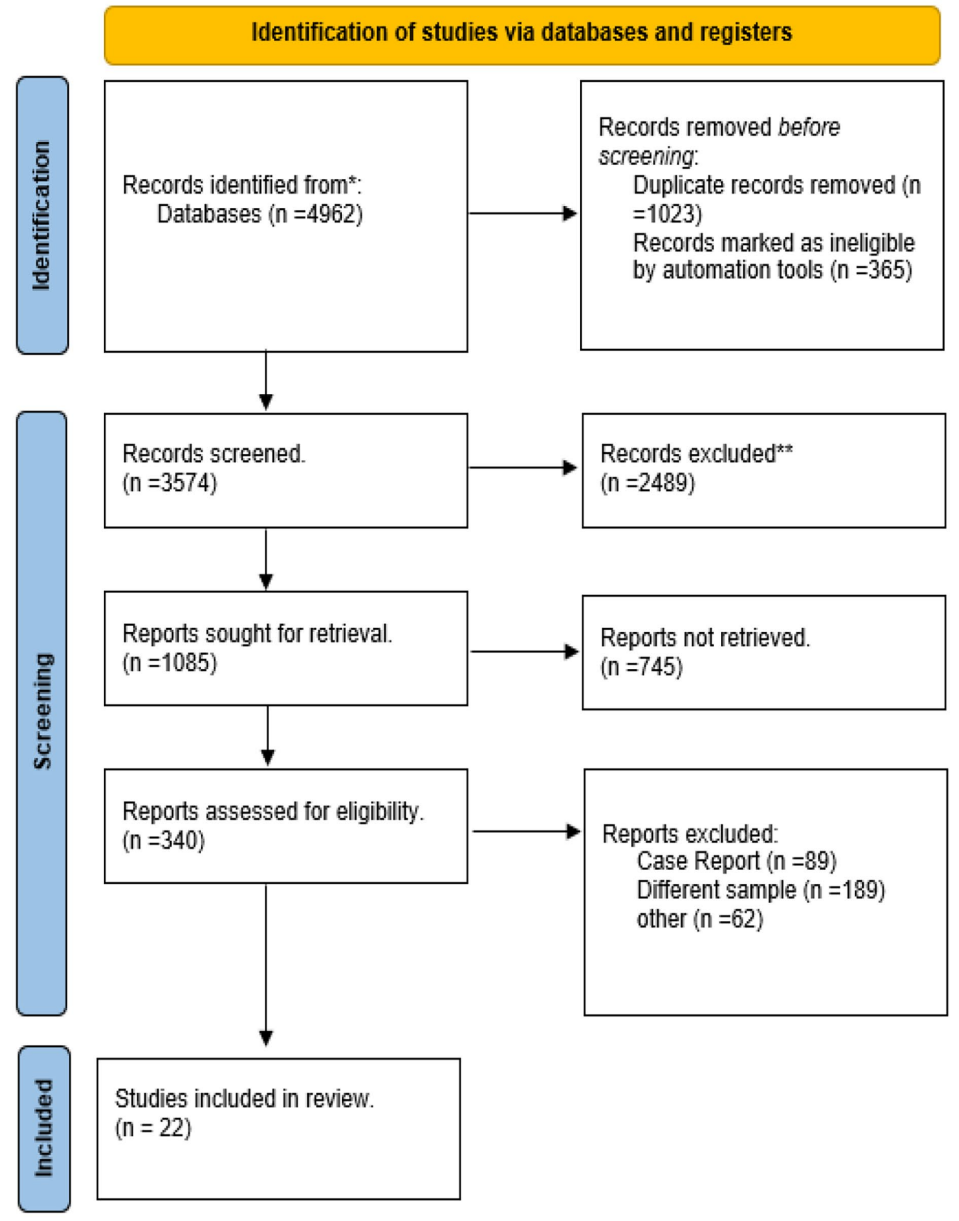
the extraction table of the included studies

We began with an initial pool of 4962 records identified through database searches. After deduplication, 3574 records remained for screening. We screened all these records and excluded 2489 based on titles and abstracts, leaving 1085 articles for full-text eligibility assessment. Upon detailed review, we excluded 745 articles for being not retrieved leaving 340 studies were assessed for eligibility, resulting in 22 studies being included in the final review [16, 45–66]. Each of these 22 studies was accounted for in our report, ensuring a comprehensive and transparent documentation of our systematic review process. as shown in Fig. 1.

### Data extraction

The review process involved two independent reviewers who screened titles and abstracts, followed by a full-text review of potentially eligible studies. Discrepancies were resolved through discussion or consultation with a third reviewer .The data extraction phase was a critical component of our systematic review, which centered on exploring ethical challenges and palliative care practices in nursing, particularly in the context of end-of-life decision-making. The main objective during this stage was to systematically collect and consolidate essential information from the selected studies, focusing on how ethical dilemmas and palliative care interventions are navigated and implemented by nurses in end-of-life care scenarios.

The extraction process involved a thorough examination of each study, with particular attention to several key aspects:

- **Study Characteristics**: We gathered detailed information on the study’s design, setting, population, and sample size. This was vital for contextualizing the research, ensuring its relevance to our review, and assessing its contribution to the field of nursing ethics and palliative care.
- **Ethical Challenges and Palliative Care Interventions**: We meticulously documented the nature of ethical dilemmas encountered by nurses, the strategies employed to address these challenges, and the specifics of palliative care interventions. This included descriptions of decision-making processes, communication techniques, advanced care planning measures, and any educational or support frameworks designed to facilitate ethical and palliative care in nursing.
- **Outcome Measures**: Our data extraction also focused on identifying and noting the outcomes used to evaluate the impact of ethical and palliative care interventions on patients, nurses, and the healthcare system. These outcomes included measures of decision-making efficacy, quality of end-of-life care, patient and nurse satisfaction, psychological well-being of healthcare professionals, and any reported challenges or adverse events associated with implementing ethical decisions or palliative care practices.

In cases where data were missing, unclear, or incomplete, efforts were made to contact the original authors for additional information, thus ensuring the accuracy and completeness of our dataset.

Additionally, we scrutinized studies for potential overlaps in study populations to prevent data duplication in our analysis. Direct communication with authors was initiated when there was any uncertainty regarding the study cohorts, which played a crucial role in maintaining the integrity and reliability of our data extraction process. This detailed and diligent approach allowed us to develop a solid foundation for our systematic review, ultimately leading to meaningful insights into the ethical challenges and palliative care practices in nursing, especially at the end of life.

### Quality assessment

In our systematic review examining ethical challenges and palliative care practices in nursing, particularly focusing on end-of-life decision-making, we underscored the imperative of assessing the methodological quality and risk of bias in the included studies. This step is vital to affirm the reliability and relevance of our findings, thereby establishing a solid basis for recommendations regarding ethical and palliative care strategies in the nursing profession.

For the evaluation of study quality, we adopted a structured methodology by utilizing the ROBVIS-II tool, an enhancement over its predecessor designed to more accurately assess the risk of bias in non-randomized studies of interventions. The choice of the ROBVIS-II tool was informed by its comprehensive capability to scrutinize various dimensions of bias, making it particularly suitable for our review’s emphasis on ethical dilemmas and palliative care approaches in the context of nursing care for end-of-life patients. While the ROBVIS-II tool was used for non-randomized clinical trials, we utilized the Joanna Briggs Institute (JBI) critical appraisal tools for qualitative studies and the Cochrane Risk of Bias tool for randomized controlled trials.

Each selected study underwent an independent assessment to meticulously explore critical dimensions such as study design, selection and categorization of participants, adherence to ethical and palliative care protocols, methods for measuring outcomes, and handling of potential confounders and missing data. This detailed scrutiny was crucial in appraising the methodological robustness of the studies and pinpointing any possible biases that could affect the conclusions drawn.

To ensure the objectivity and fairness of our assessments, we rigorously addressed any discrepancies encountered during the review process. Conflicts or uncertainties regarding the quality or risk of bias of the studies were resolved through a process of consensus-building. This involved comprehensive discussions among the members of our review team, fostering a collaborative effort that culminated in a unanimous agreement on the evaluations of the studies’ quality. This approach not only reinforced the integrity of our quality assessment process but also enhanced the credibility of our systematic review’s findings on navigating ethical challenges and implementing palliative care practices in nursing at the end of life.

### Data analysis

In this extensive systematic review, which examines ethical challenges and palliative care practices in nursing at the end of life, we adopted a rigorous data analysis methodology that combines both narrative synthesis and thematic analysis. This dual approach is essential for a nuanced understanding of the complex ethical dilemma’s nurses face and the effectiveness of palliative care strategies. Below is a detailed description of how each analytical method was utilized in our study:

1. **Narrative Synthesis**: This method formed the cornerstone of our data analysis, enabling a comprehensive and systematic review of the literature [26]. Through narrative synthesis, we meticulously evaluated the ethical challenges encountered in nursing practices, particularly in end-of-life care, and the various palliative care approaches employed. This process allowed for a deep dive into the complexities and subtleties of ethical decision-making and palliative care in nursing, facilitating an understanding of the impact of different strategies on patient care, nurse-patient relationships, and overall care quality. The narrative synthesis provided a structured narrative that highlighted key patterns, gaps in the literature, and potential areas for future inquiry. It enabled us to collate and interpret findings on the effectiveness of communication strategies, the role of advanced care planning, and the impacts of ethical education and support systems on nursing practice.
2. **Thematic Analysis**: Complementing the narrative synthesis, thematic analysis was crucial for identifying recurring themes across the studies included in our review. This qualitative technique delved into the ethical and palliative care practices within nursing, uncovering central themes such as ethical dilemmas, strategies for managing moral distress, approaches to enhancing communication with patients and families, and the integration of palliative care principles into nursing education and practice. By focusing on these themes, we explored the intricacies involved in navigating ethical challenges and implementing palliative care practices. Thematic analysis shed light on the various dimensions of end-of-life care, including the barriers to and facilitators of effective ethical decision-making and palliative care, the importance of cultural competence, and the need for interprofessional collaboration. This analytical approach enhanced our comprehension of how ethical challenges and palliative care practices are approached in nursing, emphasizing the significance of tailored interventions, ongoing education, and organizational support in improving end-of-life care.

Together, narrative synthesis and thematic analysis provided a robust framework for our systematic review, offering deep insights into the ethical challenges and palliative care practices in nursing. This combined approach enabled a rich exploration of the literature, contributing to a comprehensive understanding of the current state of knowledge and identifying avenues for future research in this critical area of nursing practice.

## Results

### Risk of bias

**Fig. 2:**
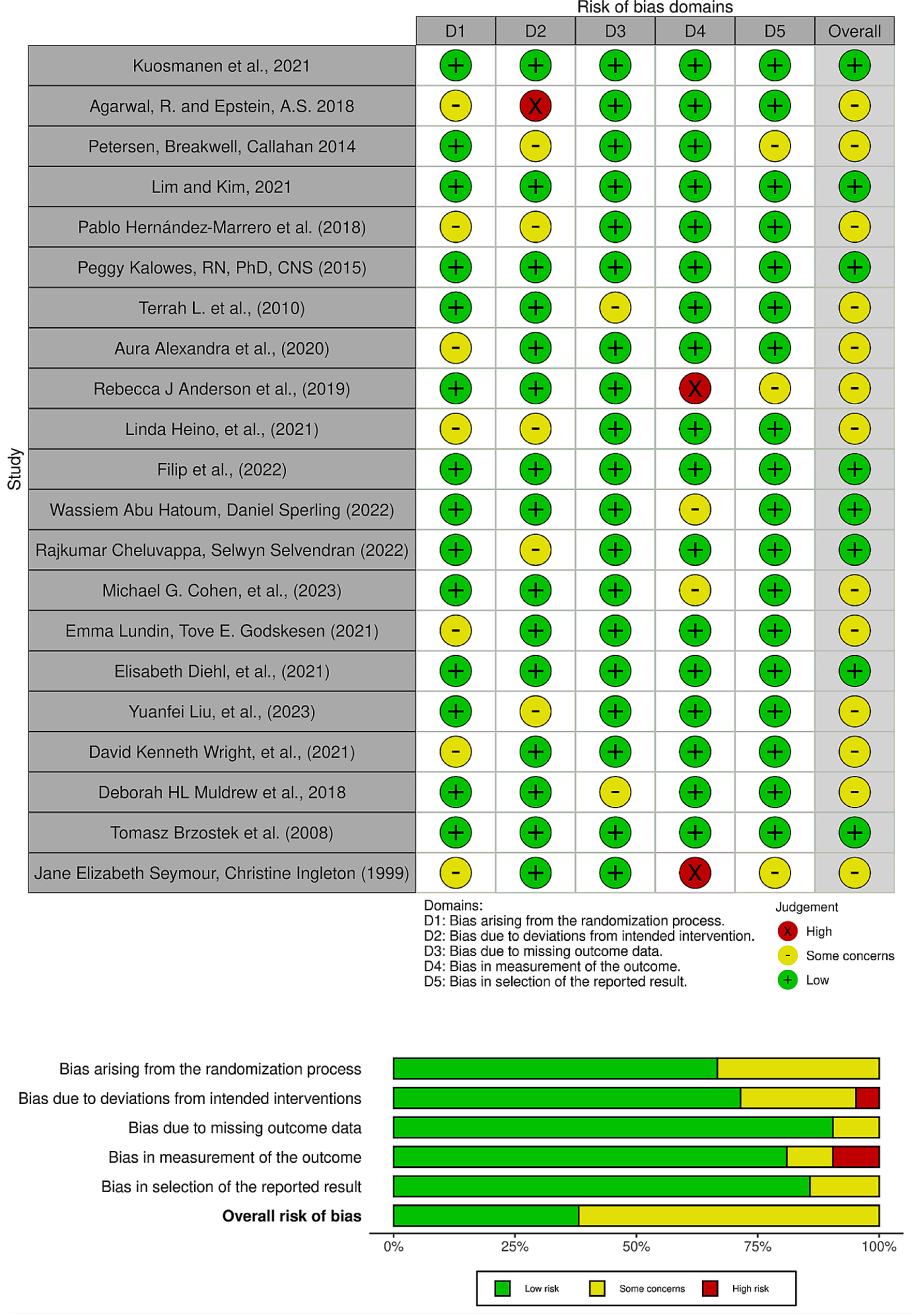
Risk of bias of the included studies

The Risk of Bias assessment of the studies listed in Fig. 2 shows a spectrum of concerns across different areas. For instance, the study by Agarwal, R. and Epstein, A.S. (2018) raises some concerns due to a high bias in the deviation from the intended intervention. In contrast, the studies by Kuosmanen et al., (2021), Lim and Kim, (2021), Peggy Kalowes (2015), Filip et al., (2022), Elisabeth Diehl et al., (2021), and Tomasz Brzostek et al. (2008) consistently show low bias in all categories, indicating a robust methodological approach.

Rebecca J Anderson et al., (2019) and Jane Elizabeth Seymour, Christine Ingleton (1999) have high bias in the measurement of outcomes, which may affect the reliability of their results. The studies by Petersen, Breakwell, Callahan (2014), Pablo Hernández-Marrero et al. (2018), Aura Alexandra et al., (2020), Linda Heino et al., (2021), and David Kenneth Wright et al., (2021) show some concerns in more than one category, but not to a degree as high as some others.

The majority of studies maintain a low bias in the selection of reported results, suggesting that reporting bias is less of a concern in this collection of studies. However, there are sporadic concerns with randomization, intended interventions, and outcome measurements that indicate areas for improvement. Overall, the studies exhibit a variable risk of bias, with many showing a good methodological quality but with some studies indicating areas that could potentially influence the outcomes and interpretations of the research.

### Main outcomes

Based on the data extracted from the studies (Table S1 ), we can elaborate on the four identified themes with comprehensive paragraphs incorporating all relevant studies:

1- **Ethical Challenges in Practice**:

- **Autonomy**: Ethical dilemmas related to patient autonomy were a prominent theme. Studies by Filip et al. (2022) and Pablo Hernández-Marrero et al. (2018) highlighted challenges in respecting patient autonomy, particularly when patients’ wishes were unclear or when surrogate decision-makers were involved. These studies underscored the importance of clear communication and advance care planning to ensure that patient preferences are respected. Additionally, decisions around providing or withholding hydration and nutrition presented significant ethical tensions, balancing patient comfort and autonomy with medical best practices.
- **Beneficence and Nonmaleficence**: The ethical implications of practices potentially hastening death, such as palliative sedation, were extensively discussed. Studies emphasized that competent palliative sedation practices aim solely at relieving intractable symptoms without the intention of hastening death (Deborah HL Muldrew et al., 2018). Nurses also faced dilemmas in balancing the benefits and harms of interventions, striving to maximize patient comfort while avoiding unnecessary suffering.
- **Justice**: Issues related to equitable access to palliative care services were significant, especially for marginalized and underserved populations. Studies reflected on the ethical intricacies of ensuring fair access to high-quality end-of-life care, highlighting the need for policies and practices that promote justice in healthcare.

2- **Communication and Decision-Making**:

- **Communication Strategies**: Effective communication is a cornerstone in end-of-life care, facilitating shared decision-making and honoring patient autonomy. The studies by Kuosmanen et al. (2021), Agarwal & Epstein (2018), and Rebecca J Anderson et al. (2019) all highlighted the importance of involving patients in decision-making processes with a strong emphasis on clear communication and understanding patient values. These studies suggested that open communication and interdisciplinary teamwork are prerequisites for meaningful participation in care decisions.
- **Interventions**: Specific communication interventions, such as training programs and structured communication models, were highlighted for their role in improving end-of-life discussions. Lim and Kim (2021) pointed towards the need for improved ethics education to enhance moral sensitivity and ethical decision-making abilities. Emma Lundin & Tove E. Godskesen (2021) and Elisabeth Diehl et al. (2021) underscored the communication barriers that exist, particularly in the care of residents with dementia, suggesting the development of better communication training programs. Together, these studies advocated for interventions to optimize communication strategies and patient education, aiming to enhance shared decision-making in palliative care settings.

3- **Integration of Palliative Care Principles and Practices**:

- **Communication Strategies**: The integration of palliative care principles into nursing practice is crucial for improving the quality of end-of-life care. Studies by Peggy Kalowes (2015), Terrah L. et al. (2010), and Aura Alexandra et al. (2020) discussed the role of nurses in leading conversations about disease trajectory and goals of care, managing family effects, culture, spirituality, communication, and ethics. These studies emphasized the need for consistent communication and early involvement in palliative care to better manage symptoms and family dynamics.
- **Nursing Leadership**: Nursing leadership was highlighted as essential in facilitating palliative care principles and advocating for improved guidelines and support systems. The utility of prognostic tools and the importance of flexibility in care plans were underscored, emphasizing the role of nurses in integrating palliative care principles into practice and ensuring that care aligns with patient values and priorities.

4- **Educational and Organizational Support**:

- **Interventions for Nurse Development**: Education and organizational support are pivotal in equipping nurses to manage the complexities of end-of-life care. Studies such as those by Linda Heino et al. (2021), Wassiem Abu Hatoum & Daniel Sperling (2022), Yuanfei Liu et al. (2023), and David Kenneth Wright et al. (2021) discussed the roles and attitudes of nurses regarding palliative sedation, shared decision-making, and coping with ethical conflict. These studies called for more research on the impact of various educational and support interventions, evaluation of standardized training, and the exploration of cultural differences and organizational factors in end-of-life care.
- **Clarifying Practices**: It is important to note that palliative care and Medical Assistance in Dying (MAID) are distinct practices. The primary focus of palliative care is on symptom management and improving the quality of life for patients with serious illnesses, with no intention to hasten death. In contrast, MAID involves intentionally ending a patient’s life at their request to relieve suffering. These practices are fundamentally different, and conflating them could lead to misunderstandings about the goals and ethical principles underlying each approach. This distinction is crucial for developing appropriate educational and organizational support for nurses.

## Discussion

The findings of this systematic review highlight the multifaceted and complex ethical challenges that nurses encounter in providing end-of-life care. By synthesizing the evidence from diverse studies, four overarching themes emerge: the centrality of communication and decision-making, the ethical dilemmas inherent in end-of-life care practice, the integration of palliative care principles, and the need for educational and organizational support. These themes underscore the interdependent nature of ethical issues, palliative care practices, and the pivotal role of nurses in navigating this intricate terrain.

1. Communication and Decision-Making:

Effective communication is a foundational element in ensuring patient autonomy and shared decision-making in end-of-life care. Several studies in this review emphasize the importance of involving patients in decision-making processes, highlighting the need for clear communication and an understanding of patient values [67–69]. Kuosmanen et al. [55]assert that interdisciplinary teamwork, open communication, and a positive patient-healthcare professional relationship are prerequisites for meaningful participation in care decisions. Rebecca J Anderson et al. [16] offer insights into communication strategies, such as highlighting deterioration, involving patients in decision-making, tailoring information, and maintaining honesty and clarity. These findings resonate with the work of Lim and Kim [70], who advocate for improved ethics education and support services to enhance nurses’ moral sensitivity and ethical decision-making abilities in end-of-life care.

The reviewed studies underscore the need for nurses to be equipped with effective communication skills and decision-making frameworks to navigate the complexities of end-of-life care. Agarwal and Epstein [54]emphasize the importance of advance care planning (ACP) in supporting patient autonomy and suggest that nurses can facilitate ACP and primary palliative care to promote informed decision-making. Emma Lundin and Tove E. Godskesen [64] and Elisabeth Diehl et al. [46] highlight the communication barriers that exist, particularly in caring for residents with dementia, advocating for the development of better communication training programs and enhanced team collaboration.

These findings collectively reinforce the pivotal role of communication in facilitating shared decision-making and honoring patient autonomy in end-of-life care. They also highlight the need for interventions to optimize communication strategies, patient education, and the integration of ethical principles into nursing practice.

2. Ethical Challenges in Practice:

End-of-life care is inherently fraught with ethical dilemmas, and nurses frequently face complex decisions that involve balancing principles of autonomy, beneficence, nonmaleficence, and justice [71–74]. Filip et al. [52]and Pablo Hernández-Marrero et al. [75] delve into the ethical decision-making involved in end-of-life care, underscoring the value of patient autonomy and the relational and organizational challenges that arise, particularly in nursing home settings. Deborah HL Muldrew et al. [63] reveal that relational issues, especially those involving residents and families, are among the most frequent and distressing ethical challenges encountered by nurses in nursing homes.

These studies reflect the intricate ethical landscape that nurses navigate, highlighting the need for nurses to be equipped with robust ethical decision-making frameworks and support systems. Petersen, Breakwell, and Callahan [59] emphasize the importance of integrating palliative care principles and ethical considerations into nursing practice, particularly in orthopedic settings, where end-of-life care may be overlooked. These findings underscore the significance of providing multidisciplinary education and fostering organizational cultures that prioritize ethical decision-making in end-of-life care across diverse healthcare settings [76, 77].

3. Integration of Palliative Care Principles and Practices:

The integration of palliative care principles into nursing practice is crucial for improving the quality of end-of-life care and addressing ethical challenges [78, 79]. Studies by Peggy Kalowes [45], Terrah L. et al. [47], and Aura Alexandra et al. [58] discuss the pivotal role of nurses in leading conversations about disease trajectories, goals of care, managing family dynamics, addressing cultural and spiritual needs, and anticipating end-of-life issues. These studies highlight the importance of consistent communication, early involvement in palliative care, and the utility of prognostic tools and flexible care plans to better manage symptoms and support families.

The findings underscore the significance of nurses embracing their leadership role in facilitating palliative care principles and advocating for improved guidelines, support systems, and organizational cultures that prioritize holistic, patient-centered care. By incorporating palliative care principles into their practice, nurses can better navigate ethical dilemmas, enhance communication, and ensure that care aligns with patients’ values and priorities throughout the end-of-life journey.

4. Educational and Organizational Support:

Equipping nurses with the necessary knowledge, skills, and support is pivotal in addressing the ethical challenges and complexities of end-of-life care [80, 81]. Several studies highlight the need for developing training programs, increasing interprofessional shared decision-making, and providing emotional and practical support for nurses [82–84].

Linda Heino et al. [56] and Wassiem Abu Hatoum and Daniel Sperling [60] emphasize the importance of educational interventions and interprofessional shared decision-making in enhancing nurses’ competencies and practices related to palliative sedation and end-of-life care for patients with end-stage renal disease (ESRD). David Kenneth Wright et al. [65]underscore the need for support and recognition of the moral identity work that palliative care nurses undergo when navigating the intersection of palliative care and medical assistance in dying (MAID).

Additionally, studies by Yuanfei Liu et al. [62] and Rajkumar Cheluvappa and Selwyn Selvendran [66] explore the coping strategies and perspectives of nurses in intensive care settings, highlighting the need for training programs and interventions to address the ethical conflicts and challenges encountered in these contexts.

These findings emphasize the importance of comprehensive educational initiatives, organizational support systems, and the fostering of collaborative, interdisciplinary approaches to equip nurses with the necessary competencies and coping mechanisms to navigate the ethical complexities of end-of-life care [85]. Furthermore, the studies suggest the need for longitudinal research to evaluate the impact of standardized training programs, organizational changes, and multidisciplinary interventions in enhancing ethical decision-making and palliative care practices across diverse healthcare settings [86, 87].

While the reviewed studies provide valuable insights into ethical challenges and palliative care practices, it is essential to acknowledge the role of cultural factors and address the disparities that exist in accessing and experiencing end-of-life care. Several authors, including Rebecca J Anderson et al. [16], Agarwal and Epstein [54], and Tomasz Brzostek et al. [61], underscore the influence of cultural beliefs, norms, and values on perceptions of end-of-life care, decision-making processes, and attitudes towards concepts such as euthanasia.

Tomasz Brzostek et al. [61] highlight the influence of personal philosophy of life and the importance of legal frameworks in shaping attitudes towards euthanasia among nurses. These findings emphasize the need for culturally sensitive approaches that respect diverse perspectives and belief systems while promoting ethical decision-making and patient-centered care.

Furthermore, studies such as Jane Elizabeth Seymour and Christine Ingleton [48] draw attention to the unique ethical challenges that arise in qualitative research involving critically ill individuals, informal carers, and healthcare staff in palliative care settings. They emphasize the importance of adopting a holistic and context-based approach, maintaining a non-judgmental attitude, practicing transparent communication, and fostering collaboration between researchers and participants.

To address the disparities and ensure equitable access to quality end-of-life care, there is a pressing need for further research on cultural competence interventions and strategies to bridge the gap between different cultural and socioeconomic backgrounds [88, 89]. Diversifying the palliative care and hospice workforce and promoting cultural humility in end-of-life services can contribute to reducing these disparities and enhancing the overall quality of care [90, 91].

### Limitation of the study

This review has several limitations that should be considered when interpreting the findings. First, there is potential publication bias, as we included only published studies, which may skew the results towards positive findings. Second, our review was restricted to studies published in English, potentially overlooking relevant research in other languages. Third, we excluded grey literature, such as conference abstracts, theses, and reports, which may contain valuable insights. Additionally, the included studies varied in their design, context, and quality, which could affect the generalizability of our conclusions. These limitations highlight the need for cautious interpretation and underscore the importance of further research to address these gaps.

### Future research directions

While this systematic review provides valuable insights, it also highlights several areas that warrant further investigation. Several authors, including Rebecca J Anderson et al. [1], Emma Lundin and Tove E. Godskesen [21], and Linda Heino et al. [13], emphasize the need for more research on communication strategies, cultural influences, and the experiences of healthcare professionals in different settings.

Additionally, longitudinal studies exploring the evolution of ethical reflections and practices in the context of MAID and palliative care, as suggested by David Kenneth Wright et al. [22], can deepen our understanding of the complex interplay between these domains.

Furthermore, researchers such as Michael G. Cohen et al. [8] and Rajkumar Cheluvappa and Selwyn Selvendran [23] call for evaluating the impact of standardized training programs, organizational changes, and multidisciplinary meetings on ethical decision-making and palliative care practices.

Moreover, comparative studies examining cultural variations in ethical considerations, as proposed by Jane Elizabeth Seymour and Christine Ingleton [5], and investigating the experiences and perspectives of healthcare staff in palliative care research can contribute to a more comprehensive understanding of this multifaceted issue.

Moral distress, defined as the psychological discomfort experienced when constrained from acting on one’s ethical beliefs, is a prevalent issue among nurses in end-of-life care. Addressing this distress is crucial for supporting nurses’ well-being and ethical practice [92, 93]. Future research should explore interventions to mitigate moral distress and its impact on nursing practice.

Future research should focus on several key areas to enhance our understanding and practice of palliative care in nursing. First, developing culturally tailored communication training programs is essential to address the diverse needs of patients and families from different backgrounds. Second, evaluating the long-term impacts of standardized palliative care education can provide insights into the sustainability and effectiveness of these programs. Third, exploring ethical decision-making frameworks in diverse clinical settings will help identify best practices and potential areas for improvement. Additionally, there is a need for longitudinal studies to assess the effectiveness of interventions aimed at reducing moral distress among nurses, which can significantly impact their well-being and quality of care. Further research should also investigate strategies to integrate palliative care principles across various healthcare settings, ensuring that all patients receive comprehensive, compassionate care at the end of life.

## Conclusion

This systematic review illuminates the intricate ethical challenges and palliative care practices that nurses encounter in end-of-life care. The four overarching themes – communication and decision-making, ethical challenges in practice, integration of palliative care principles, and educational and organizational support – highlight the interdependence of these elements and the pivotal role of nurses in navigating this complex terrain.

Addressing the ethical dilemmas in palliative care requires a multifaceted and targeted approach that encompasses education, communication, and systemic integration. This review highlights the complexity of ethical challenges faced by nurses, including issues related to patient autonomy, beneficence, nonmaleficence, and justice. To effectively navigate these challenges, several key strategies should be implemented.

First, developing and implementing culturally tailored communication training programs for healthcare providers is essential. These programs should focus on enhancing communication skills that respect and incorporate the cultural and personal values of patients and their families, thereby improving shared decision-making and ensuring that care aligns with patient preferences.

Second, evaluating the long-term impacts of standardized palliative care education on nursing practice is crucial. By assessing these programs over time, we can determine their effectiveness in enhancing nurses’ abilities to handle ethical dilemmas and improve patient outcomes. Continuous education and training in ethical decision-making, communication strategies, and palliative care principles are necessary to equip nurses with the skills required to provide high-quality end-of-life care.

Third, creating ethical decision-making frameworks that can be applied across diverse clinical settings will provide nurses with structured guidance to navigate complex ethical issues. These frameworks should be adaptable to various contexts, ensuring that nurses can make informed and consistent ethical decisions regardless of the setting.

Fourth, conducting longitudinal studies to assess the effectiveness of interventions aimed at reducing moral distress among nurses is imperative. Moral distress, which arises when nurses are unable to act according to their ethical beliefs, can lead to burnout and negatively impact patient care. Interventions designed to alleviate moral distress, such as support groups, counseling services, and resilience training, should be evaluated for their long-term benefits.

Finally, integrating palliative care principles more comprehensively into various healthcare settings will ensure that all patients receive holistic, compassionate care. This integration involves incorporating palliative care into routine clinical practice, enhancing access to palliative care services, and promoting interdisciplinary collaboration. By embedding palliative care principles throughout healthcare systems, we can ensure that end-of-life care is patient-centered, ethically sound, and focused on improving quality of life.

In conclusion, addressing the ethical dilemmas in palliative care requires a comprehensive approach that includes targeted education, effective communication, ethical frameworks, support for nurses, and systemic integration of palliative care principles. By implementing these strategies, we can enhance the ability of nurses to navigate ethical challenges and provide compassionate, high-quality care to patients at the end of life. Further research and continued efforts are needed to refine these approaches and ensure their successful implementation across diverse healthcare settings.

### Electronic supplementary material

Below is the link to the electronic supplementary material.

Supplementary Material 1

## Data Availability

The datasets generated during and/or analyzed during the current study are available from the corresponding author on reasonable request.
