# Supplementary material for: Navigating end-of-life decision-making in nursing: a systematic review of ethical challenges and palliative care practices
Source: BMC Nurs. 2024 Jul 9;23:467. doi: 10.1186/s12912-024-02087-5 (PMC11232160; doi:10.1186/s12912-024-02087-5)
Supplement: Supplementary file 1 — Supplementary Material 1 [file 12912_2024_2087_MOESM1_ESM.docx]

Table S1.. The extraction Table of the included studies

| **Author and year** | **Country** | **Study Design** | **Sample Size** | **Population Characteristics** | **Ethical Challenges Addressed** | **Palliative Care Practices** | **Key Findings** | **Themes Identified** | **Implications for Practice** | **Suggestions for Future Research** |
| --- | --- | --- | --- | --- | --- | --- | --- | --- | --- | --- |
| Kuosmanen et al., 2021[63] | Multiple (USA, Australia, the Netherlands, New Zealand, Norway, Sweden, Canada, Belgium, UK) | Integrative Review | 12 articles | Patients in palliative care, including those diagnosed with cancer, COPD, and other diseases | Shared decision-making, patient autonomy | Everyday nursing care decisions, treatment-related medical decisions, end-of-life decisions | Patients participate and desire to participate in a wide range of decision-making aspects, including everyday nursing care, medical treatment decisions, and end-of-life care. Prerequisites for participation include interdisciplinary teamwork, open communication, a good patient–healthcare professional relationship, a favorable environment, and mutual information. | Everyday nursing care decisions, treatment-related medical decisions, end-of-life decisions | The study emphasizes the importance of involving palliative care patients in decision-making processes and suggests that healthcare professionals and organizations have a primary role in enabling this participation. | Further research is needed from the patient's perspective to explore the meaning and impact of participating in shared decision-making in palliative care settings. |
| Agarwal, R. and Epstein, A.S. 2018[62] | USA | Review Article | N/A | Patients with cancer | Advance care planning, patient autonomy, and communication barriers | Integration of palliative care principles, patient-centered and values-focused care models | ACP is fundamental for supporting the personhood of patients with cancer, emphasizing the need for clear communication and understanding of patient values. | Communication strategies, patient-centered care, standardization of ACP | Nurses can facilitate ACP and primary palliative care to support informed decision-making. | Further research is needed to optimize ACP and palliative care integration, focusing on communication and patient education. |
| Petersen, Breakwell, Callahan 2014[66] | USA | Review | N/A | Orthopaedic patients | Ethical dilemmas in palliative and end-of-life care | Multidisciplinary approach, patient-centered care | Highlighted the importance of integrating palliative care principles and ethical considerations in orthopaedic nursing. | Justice, respect for persons, nonmaleficence, and beneficence | Nurses must navigate ethical dilemmas effectively and advocate for the integration of palliative care principles in orthopaedic settings. | Further research on effective strategies for implementing ethical and palliative care principles in orthopaedic nursing practice. |
| Lim and Kim, 2021[61] | South Korea | Cross-sectional descriptive survey | 171 | Nurses caring for terminal patients in a university hospital | Ethical decision-making in end-of-life care | Not specifically mentioned | Nurses' moral sensitivity and ethical decision-making abilities were measured, revealing a need for improved ethics education and support services to enhance ethical decision-making in end-of-life care. | Moral sensitivity, professional accountability, moral reasoning/moral agency, and moral practice | The study suggests enhancing ethics education and support for nurses to improve ethical decision-making in end-of-life care, highlighting the importance of moral sensitivity. | Further research could explore the impact of various educational and support interventions on nurses' ethical decision-making abilities in different healthcare settings. |
| Pablo Hernández-Marrero et al. (2018)[64] | Portugal | Qualitative secondary analysis | 32 interviews | Nurses working in palliative care in Portugal | Autonomy, disputed/controversial issues in end-of-life care | Involvement in end-of-life decision-making, assessing patients' preferences, advance care planning | Nurses value autonomy in end-of-life care and decision-making. Palliative sedation and communication are frequent disputed/controversial issues. | Autonomy, palliative sedation, communication | Nurses working in palliative care are involved in end-of-life decision-making. Further research is needed on nurses' involvement and practices. | Research on nurses’ roles and ethical challenges in different cultural and healthcare settings to enhance palliative care practices. |
| Peggy Kalowes, RN, PhD, CNS (2015)[45] | United States | Review of literature | NA | Critically ill patients with advanced progressive illnesses nearing end of life | Improving communication about prognosis, goals of care, and end-of-life issues | Role of advanced practice nurses in leading conversations about disease trajectory, prognostic scoring, goals of care; empowering nurses with knowledge to improve quality of end-of-life care | Barriers to prognostic discussions include physician hesitancy and lack of open communication; tools like prognostic scoring systems and understanding illness trajectories can help improve accuracy of discussions; early palliative care involvement is important | Importance of consistent, open communication across disease trajectory; opportunities for nurses/APRNs to lead these discussions; using tools like prognostic scoring and understanding trajectories can improve quality of discussions | Nurses/APRNs should take leadership role in communicating prognostic information and facilitating goals of care discussions; understanding trajectories, prognostic systems, functional status can empower them to do so | Assessing impact of interventions to improve consistency and accuracy of prognostic discussions; evaluating effects of early palliative care involvement on outcomes; exploring most effective communication strategies across settings/cultures |
| Terrah L. et al., (2010)[55] | United States | Literature review | N/A | Children with cancer and their families cared for from diagnosis to cure or end of life | Achieving goals of palliative care through promoting communication, culture/spirituality, ethics, and managing symptoms | Various care delivery models; managing family effects, culture/spirituality, communication, ethics; anticipating end-of-life issues | Barriers include physician hesitancy, lack of communication; prognostic tools and understanding trajectories improves discussions; early palliative care involvement is important | Importance of consistent communication across illness; nurses' role in facilitating discussions using tools like prognostic systems; anticipating end-of-life issues like symptoms, hopefulness, parenting, legacy-making | Nurses should take a leadership role in communicating prognostic information and facilitating goals of care discussions using appropriate tools | Further research on symptom distress, hopefulness, parenting experiences, continuing bonds; assessing impact of improving consistency and accuracy of prognostic discussions; evaluating effects of early palliative care involvement |
| Aura Alexandra et al., (2020)[65] | Italy | Qualitative study using focus group methodology | 41 nurses (25 RNs, 16 NAs) across 7 facilities | Nurses (RNs and NAs) caring for patients at end of life, mainly with cancer | N/A | Timing and decision-making processes around adjusting end-of-life care plans | Care plan adjustments based on detection of deterioration and continuous changes; strategies include weighing interventions, advocating for patients, sharing decisions, involving family, allowing flexibility | "When" (detecting turning points, continuous changes) and "How" (weighing interventions, advocating, sharing decisions, involving family, flexibility) to adjust care plans | Understanding implicit decision-making can improve quality of end-of-life care by preventing delays | Exploring influence of culture; comparing novice and experienced nurses; considering other health conditions; increasing sample diversity |
| Rebecca J Anderson et al., (2019)[17] | Multiple countries (USA, UK, Japan, Sweden, Canada, Australia, Hong Kong) | Qualitative systematic review | 31 articles | Healthcare professionals and bereaved relatives | N/A | Communication about prognosis and end-of-life care between HCPs and relatives | Common communication strategies include highlighting deterioration, involvement in decision-making, interactional work, tailoring, honesty/clarity, information delivery techniques, HCP roles | Highlighting deterioration, involvement in decision-making, post-decision interactional work, tailoring, honesty and clarity, specific techniques, HCP roles | Provides recommendations to help HCPs apply strategies | More research on communication in palliative care settings, with different HCPs, exploring culture/context, quantitative testing of communication interventions |
| Linda Heino, et al., (2021)[46] | Multiple (studies included from 10 countries) | Scoping review | Not applicable (not a primary study) | Nurses | Not applicable | Practices and attitudes of nurses regarding palliative sedation | Nurses have key role in palliative sedation. Attitudes are generally positive but ethical concerns exist. Communication strategies are used. | Practices, attitudes, communication strategies | Understanding nurses' perspectives can improve quality of care | More research on different settings, compared novice vs experienced nurses, other HCPs, influences of culture |
| Filip et al., (2022)[60] | Croatia | Qualitative study using focus group methodology | 20 physicians and 21 nurses (8 focus groups total) | Physicians and nurses working in NICUs and PICUs in tertiary healthcare institutions | End-of-life decision-making | End-of-life care and decision-making | Shared perspectives on end-of-life issues with differences in emphasis between professional groups and units | End-of-life process, decision-making, procedures, emotional impacts, ICU environment | Improved guidelines acknowledging nurses' roles and increased support | More research comparing settings and exploring cultural differences |
| Wassiem Abu Hatoum, Daniel Sperling (2022)[47] | Israel | Cross-sectional quantitative study using questionnaires | 89 nephrology nurses | Nephrology nurses working in hospitals | End-of-life decision-making in ESRD patients | Shared decision-making and educational training in end-of-life care | Views and practices of SDM and training in EOL care; influences of attitudes, experience and demographics | SDM process and training; barriers and facilitators | Increase interprofessional SDM and training in EOL care | Further study of influences of experience, demographics and organizational factors; evaluation of standardized training |
| Rajkumar Cheluvappa, Selwyn Selvendran (2022) [53] | Australia | Qualitative study using focus groups | 15 physicians, 15 nurses | Physicians and nurses working in NICUs and PICUs | End-of-life decision-making | End-of-life care and decision-making | Shared perspectives on end-of-life issues with differences in emphasis between professional groups and units | Critical illness, end-of-life procedures, spill-over, four walls of ICU | Improved guidelines acknowledging nurses' roles, increased support | Further study of influences of experience, demographics and organizational factors; evaluation of standardized training |
| Michael G. Cohen, et al., (2023) [59] | United States | Secondary analysis of qualitative data (interviews and field notes) | Four qualitative datasets, including 44 interviews and 9 team observation field notes | Nephrology nurses | End-of-life decision-making in ESRD patients | Shared decision-making and educational training regarding end-of-life care | Identification of barriers and facilitators to SDM in end-of-life care according to healthcare practitioners | SDM process; training; emotional and practical support; decision-making; NICU environment | Further exploration of cultural differences, legal frameworks, organization of care and support for healthcare professionals | Evaluate impact of standardized training, organizational changes and multidisciplinary meetings |
| Emma Lundin, Tove E. Godskesen (2021)[51] | Sweden | Qualitative study using semi-structured interviews | 13 nurses | Nurses working in nursing homes | End-of-life care for residents with advanced dementia and pain | End-of-life care; communication; relational and organizational challenges | Communication, decision-making and ethical issues in end-of-life care for residents with dementia | Communication barriers; challenges in relational and organizational structures | Improve communication training and team collaboration to enhance end-of-life care | Explore differences in views between urban and rural areas and international comparisons |
| Elisabeth Diehl, et al., (2021)[54] | Germany | Nationwide cross-sectional survey | 437 nurses in GPC, 1316 nurses in SPC | Nurses working in GPC and SPC | End-of-life care for residents with advanced dementia and pain | Communication, relational and organizational challenges | Differences in burdens, resources, health, and wellbeing between GPC and SPC nurses | Communication barriers; challenges in relational and organizational structures | Develop training programs for nurses to address identified issues and implement specialist nursing roles | Explore influences of experience and organizational factors; longitudinal intervention studies |
| Yuanfei Liu, et al., (2023)[57] | China | Qualitative study using semi-structured interviews | 15 intensive care nurses | Intensive care nurses | Coping with ethical conflict in clinical practice | End-of-life care practices in ICU | Detachment and engagement coping strategies including ignoring problems, seeking emotional expression, perspective-taking, and identifying positives | Communication barriers; challenges in relational and organizational structures | Develop training programs to address identified issues | Explore influences of experience and organizational factors; qualitative and quantitative longitudinal studies |
| David Kenneth Wright, et al., (2021)[52] | Canada | Qualitative study | 22 | Palliative care nurses | Intersection of palliative care and medical assistance in dying (MAiD) | Navigating the demarcation between palliative care and MAiD in everyday practice | Most participants engaged in an ongoing process of questioning and self-examination regarding the compatibility of MAiD with palliative care. Nurses grappled with the finality of MAiD.  Balancing non-abandonment and respecting individuals' choices. | Nuanced ethical reflections on the compatibility of MAiD and palliative care  Grappling with the finality of MAiD.  Balancing non-abandonment and respecting individuals' choices. | Support for palliative care nurses in navigating ethical tensions related to MAiD.  Recognition of the moral identity work that MAiD catalyzes among palliative care nurses. | Exploration of experiences and perspectives of other healthcare professionals in end-of-life care.  Longitudinal studies on the evolution of ethical reflections and practices in the context of MAiD and palliative care. |
| Deborah HL Muldrew et al., 2018[50] | United Kingdom | an exploratory, sequential, mixed-methods design. | 69 registered nurses and 129 healthcare assistants from 18 nursing homes | The participants were registered nurses (RNs) and healthcare assistants (HCAs) working in nursing homes. | The study explored ethical issues in practice, relational issues, and organizational issues related to palliative care provision in nursing homes. | The focus of the study was on palliative care provision in nursing homes. | The study revealed that relational issues, particularly those involving residents and families, were the most frequent and caused the greatest distress. Three main themes emerged from the data: ethical issues in practice, relational issues, and organizational issues. The shared environment was identified as a key factor in the experience of ethical challenges. | efforts should be made to improve staff knowledge and enhance service organization to address ethical challenges effectively. | The findings highlight the importance of providing multidisciplinary education to enhance ethical decision-making in palliative care settings | Further research is needed to delve deeper into ethical issues in palliative care provision in nursing homes. |
| Tomasz Brzostek et al. (2008)[48] | Poland | Exploratory study | 206 graduates, 252 experienced nurses | Recently graduated nurses with a bachelor's degree, experienced nurse practitioners | Perception and understanding of palliative care and euthanasia | Not specifically focused | - High subjective perception and understanding of palliative care and euthanasia terms.  - Exclusion of euthanasia from palliative care.  - Influence of personal philosophy of life on attitudes towards euthanasia.  - Importance of the law in shaping attitudes towards euthanasia. | - Perception and understanding of palliative care and euthanasia  - Exclusion of euthanasia from palliative care  - Influence of personal philosophy of life - Importance of the law | Understanding factors influencing nurses' attitudes towards euthanasia and palliative care can guide appropriate care and support. | - Explore the impact of education and training on nurses' perceptions  - Investigate the influence of cultural and religious factors on attitudes and practices.  - Investigate the experiences and perspectives of terminally ill patients and their families. |
| Jane Elizabeth Seymour, Christine Ingleton (1999)[56] | Not specified | Qualitative research | Not specified | Critically ill individuals, informal carers, healthcare staff | - Ensuring autonomous choice and informed consent  - Avoiding harm to participants  -Maintaining confidentiality and anonymity  - Respecting and preserving dignity of participants | Exploring and evaluating palliative care services | - Qualitative research in palliative care presents unique ethical challenges  - Ethical implications should be considered throughout the research process  - Challenges related to gaining access and consent  - Dilemmas associated with preserving anonymity | - Ethical complexities in research with dying individuals and their carers  - Importance of research design in establishing an ethical framework  - Managing the researcher's role during fieldwork  - Preserving anonymity in research reporting | - Adopt a holistic and context-based approach in studying individuals and groups in palliative care settings  - Emphasize the importance of research process in addition to findings  - Maintain a non-judgmental attitude and respect participants' rights and perspectives  - Practice transparent and honest communication about the research  - Foster collaboration between researchers and participants | - Further exploration of ethical challenges in qualitative research in palliative care - Additional strategies and guidelines for addressing ethical concerns - Longitudinal studies on the impact of qualitative research - Comparative studies on cultural variations in ethical considerations - Research on experiences and perspectives of healthcare staff in palliative care research |
